# Sex differences in metabolic remodeling and skeletal muscle regeneration following cardiotoxin-induced injury

**DOI:** 10.1038/s41598-026-62000-9

**Published:** 2026-07-13

**Authors:** Jong Beom Jin, Tori Soukup, Angelique Robinson, Ember Black, Addison Williams, Atul Pranay, Kenneth Humphries, Dong Yeon Kim, Yoo Kim, Edralin Lucas, Shane M. Hammer, Jiyoung Bae

**Affiliations:** 1 Department of Nutritional Sciences, Oklahoma State University, 420 Nancy Randolph Davis, Stillwater, OK 74078 USA; 2 Metabolic Phenotyping Core, Aging & Metabolism Research Program, Oklahoma Medical Research Foundation, Oklahoma City, OK USA; 3 Department of Food Science and Nutrition, Gyeongkuk National University, Andong, Gyeongsangbuk-Do Republic of Korea; 4 School of Kinesiology, Applied Health, and Recreation, Oklahoma State University, Stillwater, OK USA

**Keywords:** Skeletal muscle regeneration, Sex differences, Glycolysis, Metabolomics, Molecular biology, Physiology

## Abstract

Skeletal muscle regeneration is a dynamic biological process that requires metabolic remodeling, satellite cell activation, inflammatory responses, and tissue remodeling. Although biological sex is recognized as an important determinant of skeletal muscle regeneration, whether males and females utilize distinct metabolic programs during regeneration remains poorly understood. In the present study, we investigated sex-dependent differences in metabolic remodeling and regenerative responses following cardiotoxin (CTX)-induced skeletal muscle injury in mice. Targeted metabolomic analysis performed at 7 days post-injury (DPI) revealed clear sex-dependent metabolic remodeling. Principal component analysis and pathway enrichment analysis identified glycolysis/gluconeogenesis as the most significantly altered metabolic pathway following injury. Male mice exhibited higher levels of several glycolytic, tricarboxylic acid (TCA) cycle, and amino acid metabolites than female mice. In contrast, female mice showed greater expression of Pax7, Myf5, and Myod1, together with increased Pax7-positive cells, greater Cyclin D1 staining, and higher expression of Ccne1, Cdc2, and Cdk4. Local inflammatory responses also differed between sexes, with distinct temporal patterns of MCP-1, IL-6, TNF-α, and CD45 during regeneration. Histological analyses demonstrated greater lipid accumulation in female muscle at 7 DPI, whereas both sexes exhibited comparable percentages of centrally nucleated fibers and similar restoration of muscle architecture by 21 DPI. Together, these findings indicate that biological sex is associated with differences in metabolism, satellite cell-associated responses, inflammatory responses, and tissue remodeling during skeletal muscle regeneration. Although males and females ultimately achieved comparable structural recovery, the regenerative process differed substantially during the early phase after injury. These findings provide additional insight into the biological processes underlying sex-dependent skeletal muscle regeneration and establish a foundation for future studies investigating the mechanisms linking metabolism to regeneration.

**Supplementary Information:**

The online version contains supplementary material available at https://doi.org/10.1038/s41598-026-62000-9.

## Introduction

Skeletal muscle accounts for approximately 40% of total body mass and plays essential roles in locomotion, whole-body metabolism, and glucose homeostasis^1^. Following injury caused by trauma, toxins, disease, or aging, skeletal muscle has a remarkable capacity for regeneration. This process depends on satellite cell activation, inflammatory responses, and tissue remodeling to restore muscle structure and function^1^. Impaired regeneration contributes to muscle weakness and functional decline in a variety of pathological conditions, emphasizing the importance of understanding the biological mechanisms that regulate skeletal muscle regeneration.

Increasing evidence indicates that metabolism is not simply a source of energy during regeneration but also an important regulator of stem cell function and tissue regeneration. Metabolic pathways influence satellite cell activation, proliferation, and differentiation through regulation of cellular signaling, biosynthetic activity, and epigenetic processes^2,3^. In particular, glucose metabolism undergoes dynamic remodeling following muscle injury, and increased glucose utilization has been associated with improved regenerative capacity^4^. These observations suggest that metabolic regulation is closely associated with skeletal muscle regeneration.

Biological sex is another important factor influencing regenerative responses. Human studies have demonstrated sex-dependent differences in satellite cell expansion and inflammatory gene expression following eccentric contraction-induced muscle injury^5^. Experimental studies have also reported sex-dependent differences in skeletal muscle adaptation, regeneration, and muscle stem cell function^6–9^. In addition, estrogen signaling has been shown to influence satellite cell activation and inflammatory responses during skeletal muscle regeneration^7^. Collectively, these studies suggest that males and females utilize distinct biological programs during regeneration.

Skeletal muscle metabolism also differs substantially between males and females under physiological conditions^8,9^, raising the possibility that sex-dependent metabolic differences contribute to differences in regenerative responses following injury. However, despite increasing recognition of both metabolism and biological sex as important regulators of skeletal muscle regeneration, it remains unclear whether metabolic remodeling during regeneration differs between males and females and how these metabolic differences are associated with regenerative responses.

Therefore, the objective of the present study was to determine whether metabolic remodeling during CTX-induced skeletal muscle regeneration differs between males and females and whether these metabolic differences are associated with satellite cell-associated responses, inflammatory responses, and tissue remodeling. By integrating targeted metabolomics with molecular, immunohistochemical, and histological analyses, we provide a comprehensive evaluation of sex-dependent regenerative responses during skeletal muscle regeneration.

## Materials & methods

### Animals and experimental design

Eight-week-old C57BL/6 J mice (Jackson Laboratory, Bar Harbor, ME) were used for all experiments. Mice were housed in groups of three per cage in a temperature- and humidity-controlled room (22 ± 1 °C, 50–60% humidity) with a 12-h light/dark cycle and given ad libitum access to standard chow and autoclaved or reverse osmosis water. All animal procedures were approved by the Institutional Animal Care and Use Committee (IACUC) of Oklahoma State University (Protocol #23–20). All experiments were performed in accordance with relevant guidelines and regulations.

Mice were assigned to male control, male injury, female control, and female injury groups. Both tibialis anterior (TA) muscles from each mouse were used for analysis. Final sample sizes ranged from 6 to 8 mice per group depending on tissue availability and assay requirements.

### Muscle injury induction and tissue collection

Muscle injury was induced by intramuscular injection of cardiotoxin (CTX; 10 µM in sterile 0.9% saline, Sigma-Aldrich) into the TA muscle of anesthetized mice. Anesthesia was maintained with 2% isoflurane in oxygen, and post-operative analgesia was provided via subcutaneous injection of sustained-release buprenorphine (Ethiqa XR, 3.25 mg/kg at 1.3 mg/mL) in the interscapular region. A total of 50 µL of CTX was divided into two 25 µL injections and administered into the upper and lower regions of the TA muscle using a 29-gauge insulin syringe. Control mice were not injected with CTX.

At 1, 3, 7, and 21 days post injury (DPI), mice were deeply anesthetized with 2% isoflurane in oxygen. Blood was collected by cardiac puncture under deep anesthesia into sodium citrate tubes. Blood samples were centrifuged at 2,000 × g for 15 min at 4 °C to separate plasma, which was collected and stored at − 80 °C until analysis. TA muscles from both hindlimbs were then carefully dissected, and euthanasia was completed by removal of the heart. Muscles were rinsed in 1 × PBS to remove debris and connective tissue and were either processed immediately or snap-frozen in liquid nitrogen and stored at − 80 °C for later analysis. Muscles designated for histological evaluation were fixed in 4% paraformaldehyde for 24 h, transferred to 70% ethanol, and embedded in paraffin blocks for sectioning.

### Metabolomic profiling

Targeted liquid chromatography–mass spectrometry (LC–MS) assays were performed using a previously described method^10,11^. Briefly, TA muscles collected at 7 DPI were snap-frozen in liquid nitrogen, pulverized, and homogenized on a tissue lyser. Metabolites were extracted with MeOH:H_2_O (80:20, v/v), and adonitol was added to each sample as an internal standard. Metabolite extracts were passed through Captiva EMR-lipid removal cartridges for removal of lipids. A 500 µL of each extract was dried in a vacuum centrifuge and reconstituted in 100 µL ACN:H₂O (70:30, v/v) for LC–MS analysis. For quality control (QC), 50 µL from each sample was pooled to generate a QC sample. Samples were randomized prior to batch analysis.

LC–MS analysis was performed on an Agilent 6546 LC/Q-TOF coupled to an Agilent 1290 Infinity II LC. Chromatographic separation was achieved on an InfinityLab Poroshell 120 HILIC-Z column (Agilent Technologies, Santa Clara, CA) using an aqueous ammonium acetate buffer and an acetonitrile buffer as the mobile phases. Hydrophilic interaction liquid chromatography (HILIC) was used with an injection volume of 1 µL. Targeted metabolomic profiling focused on glycolytic intermediates and tricarboxylic acid (TCA) cycle intermediates. Data processing, peak integration, and quantification were performed using Agilent MassHunter Quantitative Analysis software (version 10.1).

For identification of target metabolites, a custom Agilent Personal Compound Database and Library (PCDL) was generated from the Agilent METLIN PCDL and included glycolytic and TCA cycle intermediates. The retention times derived from analyses of inhouse prepared mix of analytical standards of glycolytic and tricarboxylic acid cycle intermediates (Cambridge isotope laboratories, Inc., Cat. No. MSK-CAA-US-1) were also included in the custom PCDL to facilitate the identification of these metabolites in the samples. Relative abundance values for target metabolites were obtained by normalizing the raw data with tissue weights and adonitol (added as internal standard) values. Pathway enrichment analysis was performed using MetaboAnalyst (version 6.0; https://www.metaboanalyst.ca). Significantly altered metabolites identified from the metabolomic dataset were mapped to KEGG metabolic pathways. Enriched pathways were ranked based on enrichment analysis, and the results were visualized as a bubble plot according to enrichment significance and enrichment ratio.

### Gene expression analysis (qPCR)

Total RNA was extracted from TA muscles using TRIzol reagent (Invitrogen) according to the manufacturer’s instructions. RNA purity and concentration were assessed using a NanoDrop spectrophotometer (Thermo Fisher Scientific), and 1 μg of total RNA was used for complementary DNA (cDNA) synthesis with the iScript cDNA Synthesis Kit (Bio-Rad). Quantitative PCR was performed using iTaq Universal SYBR Green Supermix (Bio-Rad) on a CFX Opus 384 Real-Time PCR System (Bio-Rad).

Expression of myogenic regulatory factors (Pax7, Myf5, Myod1, and Myog) and cell cycle-associated genes (Ccne1, Cdc2, and Cdk4) was quantified by qPCR. Reactions for myogenic regulatory factors were performed in technical triplicates, whereas reactions for cell cycle-associated genes were performed in technical duplicates. B2m (Beta-2-microglobulin) was used as the endogenous control, and relative gene expression was calculated using the 2^ − ΔΔCt method. Primer sequences are provided in Supplementary Table S1.

### Enzyme-linked immunosorbent assay (ELISA)

Inflammatory markers in TA muscle lysates and plasma were measured using commercially available ELISA kits (R&D Systems) for MCP-1 and IL-6 according to the manufacturers’ instructions. For muscle lysate preparation, TA tissues were homogenized in ice-cold PBS containing 0.1% Triton X-100 and protease inhibitors. Homogenates were centrifuged at 12,000 × g for 10 min at 4 °C, and the supernatants were collected for analysis. Total protein concentration of each lysate was determined using a bicinchoninic acid (BCA) assay for normalization. For plasma collection, whole blood was collected at necropsy into sodium citrate tubes and centrifuged to separate plasma. The plasma fraction was collected and stored at − 80 °C until analysis.

For ELISA, standards and samples were loaded into 96-well plates and processed according to the kit protocols. Absorbance was measured at 450 nm using a microplate reader. Cytokine concentrations were calculated from standard curves. Muscle lysate cytokine levels were normalized to total protein content and expressed as pg/mg protein, whereas plasma cytokine levels were expressed as pg/mL.

### Histological analysis

Paraffin-embedded TA muscle Sects. (5 μm) were deparaffinized in xylene (three changes, 3 min each) and rehydrated through graded ethanol solutions (100%, 95%, 70%) to distilled water. Sections were stained with Mayer’s hematoxylin for 10 min, rinsed in running tap water, and blued using Scott’s tap water substitute. Sections were then counterstained with eosin Y for 1 min, dehydrated through graded ethanol solutions, cleared in xylene, and coverslipped using Permount mounting medium. Images were acquired using a Keyence BZ-X710 microscope at 20 × magnification. Representative images were selected to illustrate morphological features of muscle regeneration.

### Oil red O staining

Frozen TA muscle Sects. (9 μm) were air-dried and stained using an Oil Red O Stain Kit (Newcomer Supply, USA) according to the manufacturer’s instructions. Briefly, sections were incubated in 100% propylene glycol, stained with preheated Oil Red O solution (60 °C) for 10 min, differentiated in 85% propylene glycol, and counterstained with Mayer’s hematoxylin. Sections were rinsed with tap water and mounted using an aqueous mounting medium. Images were acquired using a Keyence BZ-X710 microscope. Lipid accumulation was quantified as the percentage of Oil Red O-positive area relative to the total tissue area using ImageJ software.

### Immunohistochemistry

Frozen TA muscle Sects. (9 μm) were air-dried and fixed in 10% neutral buffered formalin. Sections were blocked and incubated overnight at 4 °C with primary antibodies against Cyclin D1 (sc-8396, Santa Cruz Biotechnology, 1:100), TNF-α (ab34674, Abcam; 1:100), or CD45 (ab10558, Abcam; 1:100). Following washing with PBST, sections were incubated with HRP-conjugated secondary antibody (CST #7074P2) for 30 min at room temperature. Immunoreactivity was visualized using DAB substrate (ab64238, Abcam) and counterstained with Mayer’s hematoxylin. Sections were mounted with Permount, and images were acquired using a Keyence BZ-X710 microscope. Positive staining area was quantified using ImageJ software.

### Immunofluorescence

Frozen TA muscle Sects. (9 μm) were air-dried and fixed in 10% neutral buffered formalin. Sections were blocked and incubated overnight at 4 °C with primary antibodies against Pax7 (Proteintech, #20,570-1-AP, 1:100) and laminin (Proteintech, #67,705-1-IG, 1:200). After washing with PBST, sections were incubated for 1 h at room temperature with Alexa Fluor 488-conjugated goat anti-rabbit IgG and Alexa Fluor 594-conjugated goat anti-mouse IgG secondary antibodies. Nuclei were counterstained with DAPI, and sections were mounted using Fluoromount-G™ Mounting Medium. Fluorescence images were acquired using a Keyence BZ-X710 fluorescence microscope. Pax7-positive cells were quantified using ImageJ software.

### Statistical analysis

All data are presented as mean ± standard error of the mean (SEM). Statistical analyses were performed using GraphPad Prism (version 9, GraphPad Software). Comparisons between two groups were conducted using unpaired two-tailed Student’s t-tests. For analyses involving both sex and experimental condition (e.g., myogenic gene expression), two-way analysis of variance (ANOVA) was performed to assess main and interaction effects. All analyses were performed at the animal level (N). qPCR results were normalized to the housekeeping gene prior to statistical testing. A *p*-value < 0.05 was considered statistically significant.

## Results

### Sex-dependent metabolic remodeling during skeletal muscle regeneration

**Fig. 1:**
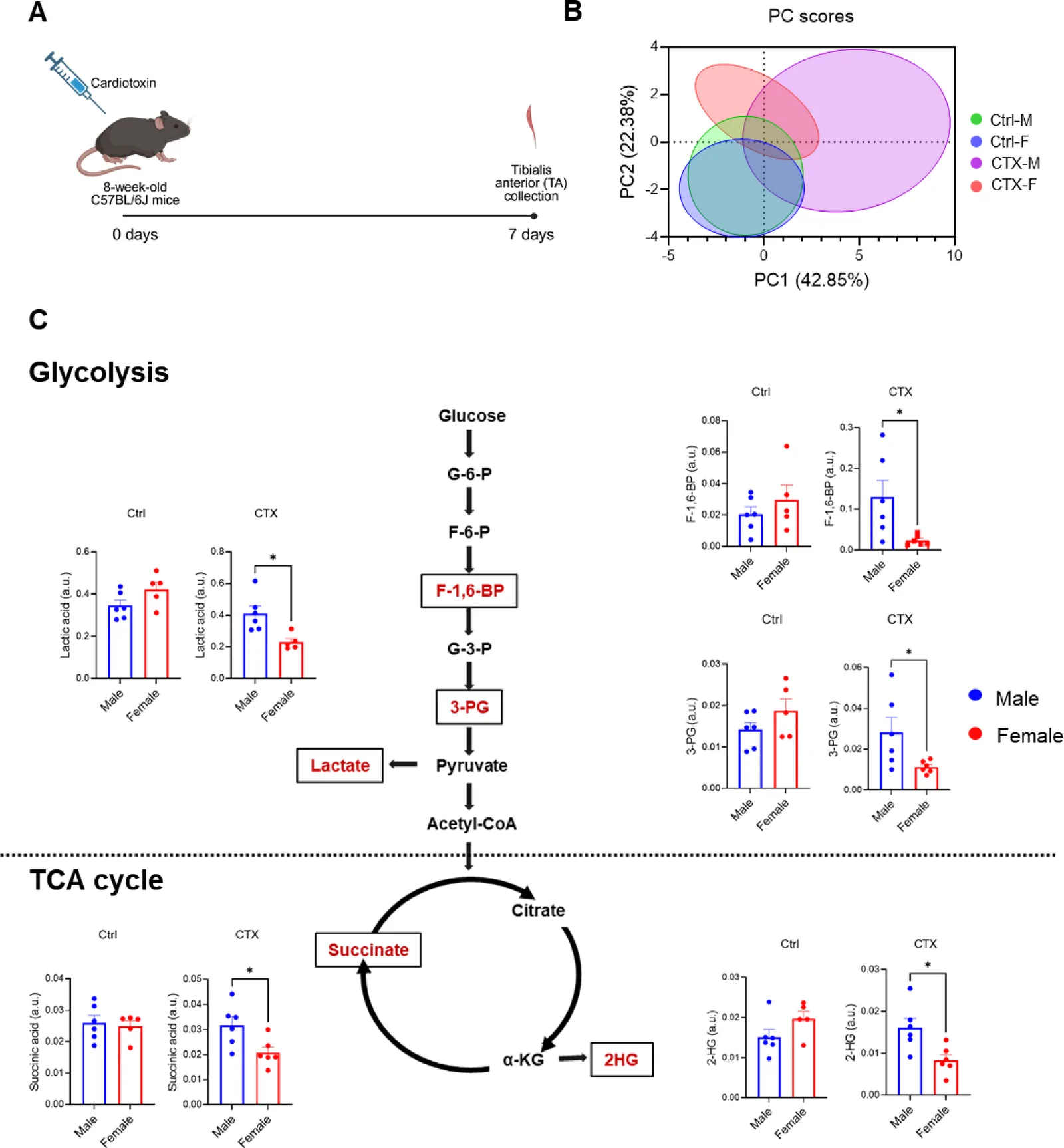
Sex-dependent metabolic remodeling during skeletal muscle regeneration. (**A**) Experimental design for targeted metabolomic analysis. Eight-week-old male and female C57BL/6 J mice received cardiotoxin (CTX) injection into the tibialis anterior (TA) muscle, and tissues were collected at 7 days post injury (DPI). (**B**) Principal component analysis (PCA) of normalized metabolite abundance in control and CTX injured TA muscle from male and female mice. (**C**) Targeted metabolomic analysis of glycolytic and tricarboxylic acid (TCA) cycle intermediates. Relative metabolite abundances are mapped onto metabolic pathways to illustrate group-specific differences. Data were obtained by LC–MS, normalized to adonitol (internal standard) and tissue weight. Each group represents n = 5–6 mice. Statistical analysis was performed using unpaired two-tailed Student’s t-tests for comparisons between males and females within each condition. *p < 0.05.

To determine whether metabolic remodeling during skeletal muscle regeneration differs between males and females, targeted metabolomic analysis was performed using tibialis anterior (TA) muscles collected at 7 days post-injury (DPI), a time point characterized by active regeneration (Fig. 1A). Principal component analysis (PCA) showed that metabolomic profiles from control muscles clustered closely regardless of sex, whereas injured muscles formed distinct clusters, indicating that metabolic differences became more pronounced following CTX-induced injury (Fig. 1B).

To identify metabolic pathways associated with these differences, pathway enrichment analysis was performed using significantly altered metabolites. Glycolysis/gluconeogenesis was identified as the most significantly enriched pathway, indicating that central carbon metabolism was differentially regulated between males and females during regeneration (Supplementary Fig. S1). We next examined individual metabolites involved in glycolysis and the tricarboxylic acid (TCA) cycle. Under control conditions, lactate, fructose-1,6-bisphosphate (F-1,6-BP), 3-phosphoglycerate (3-PG), succinate, and 2-hydroxyglutarate (2-HG) did not differ significantly between sexes. Following CTX injury, however, female mice exhibited significantly lower levels of these metabolites than male mice (Fig. 1C). Together, these findings indicate that CTX-induced injury accentuates sex-dependent metabolic differences, particularly within central carbon metabolism.

Because these metabolic differences were not restricted to glycolysis and the TCA cycle, we next investigated whether amino acid metabolism also differed between males and females during regeneration.

### Sex-dependent differences in amino acid metabolism during skeletal muscle regeneration

**Fig. 2:**
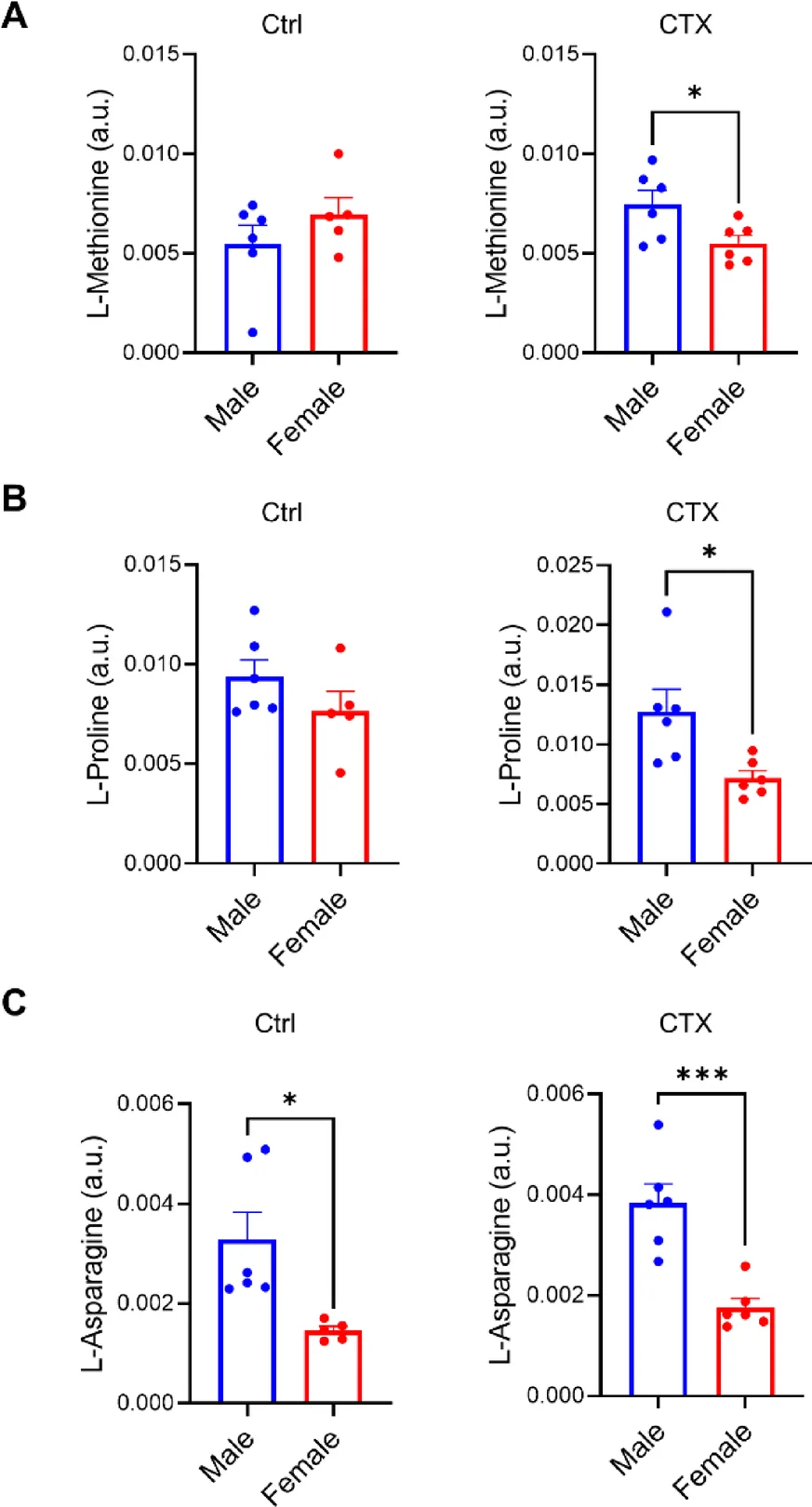
Sex-dependent differences in amino acid metabolism during skeletal muscle regeneration. Relative abundances of amino acid metabolites in tibialis anterior muscles from male and female mice at 7 days post-injury (DPI). (**A**) Methionine, (**B**) Proline, and (**C**) Asparagine. Data are presented as mean ± SEM. Statistical analysis was performed using unpaired two-tailed Student’s t-tests for comparisons between males and females within each condition. *p < 0.05, ***p < 0.001.

To further characterize metabolic changes during regeneration, we quantified selected amino acid metabolites in TA muscle collected at 7 DPI (Fig. 2). Methionine and proline levels were comparable between sexes under control conditions but were significantly higher in male mice following CTX injury (Fig. 2A and B). Asparagine levels were consistently higher in males under both control and injured conditions (Fig. 2C). These findings indicate that sex-dependent metabolic differences extend beyond central carbon metabolism to include amino acid metabolism during regeneration.

### Sex-dependent regulation of regenerative gene expression during skeletal muscle regeneration

**Fig. 3:**
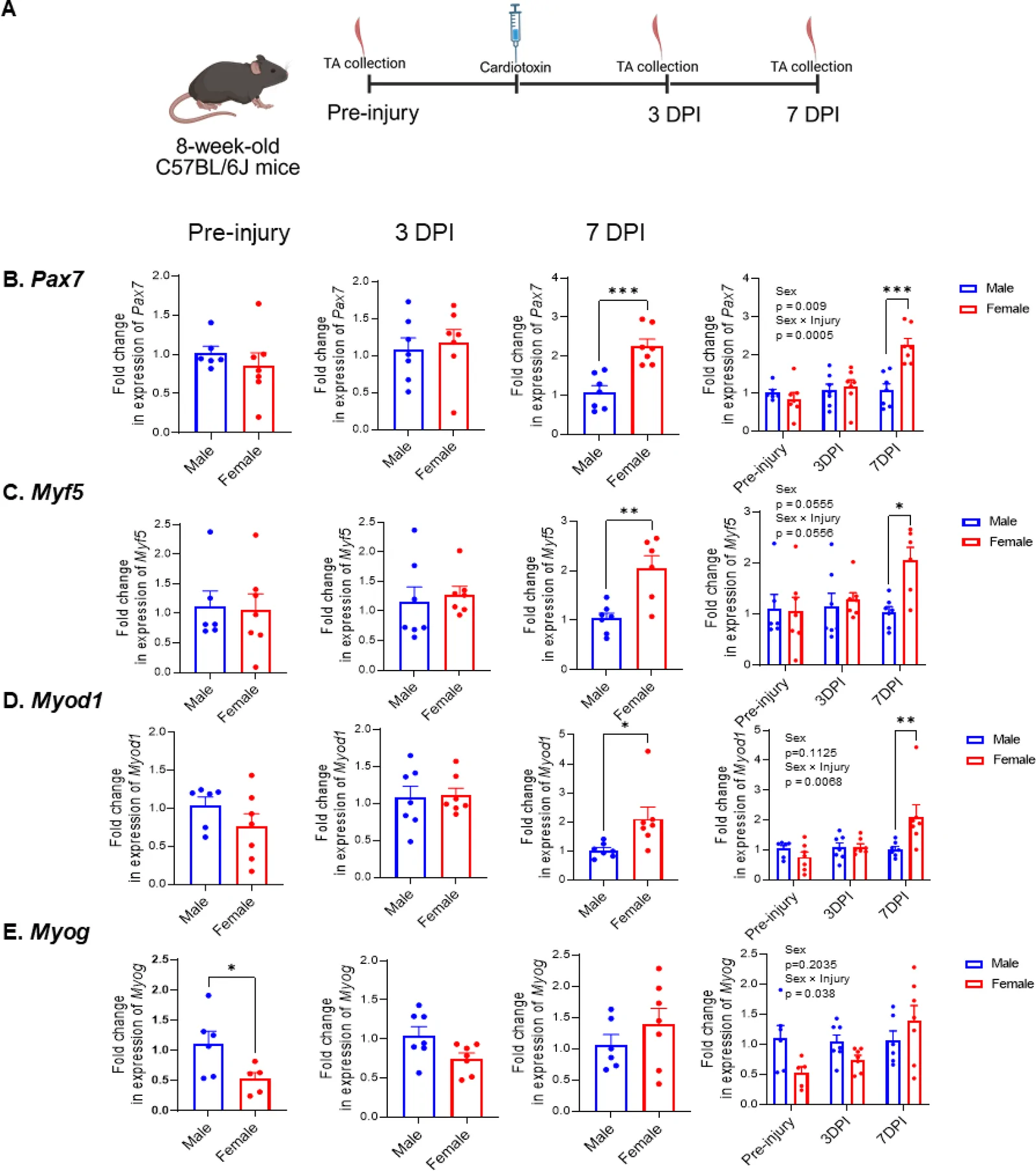
Sex-dependent regulation of regenerative gene expression during skeletal muscle regeneration. (**A**) Experimental design for gene expression analysis in TA muscle collected before injury and at 3 and 7 days post injury (DPI) after CTX injection. (**B**–**E**) Relative mRNA expression levels of myogenic regulatory factors, including *Pax7* (**B**), *Myf5* (**C**), *Myod1* (**D**), and *Myog* (**E**), in male and female mice at indicated time points. Gene expression was measured by quantitative PCR and normalized to a housekeeping gene. Data are presented as fold change relative to sex-matched pre-injury controls. Each group represents n = 7 mice. Statistical analysis was performed using unpaired two-tailed Student’s t-tests for comparisons between males and females at each time point. Two-way ANOVA was used to assess the effects of sex and injury (time), and their interaction. *p < 0.05, **p < 0.01, ***p < 0.001.

To determine whether sex-dependent metabolic differences were associated with differences in regenerative gene expression, we quantified the expression of myogenic regulatory factors before injury and at 3 and 7 DPI (Fig. 3). Gene expression was normalized to the corresponding sex-matched control group. At baseline and 3 DPI, expression of most myogenic markers did not differ significantly between males and females. In contrast, female mice exhibited significantly greater expression of Pax7, Myf5, and Myod1 than male mice at 7 DPI (Fig. 3B–D). Myog displayed a different pattern, with higher basal expression in males but no significant sex difference following injury (Fig. 3E). Two-way ANOVA further supported these findings. Pax7 showed significant effects of sex and sex × injury interaction, whereas Myod1 also exhibited a significant interaction effect. Although Myog demonstrated a significant interaction, expression levels converged following injury. Together, these findings indicate that female mice exhibit greater activation of the regenerative transcriptional program during the intermediate stage of skeletal muscle regeneration.

Because Pax7 is closely associated with satellite cell activation, we next examined satellite cell-associated and cell cycle-associated responses during regeneration.

### Sex-dependent satellite cell-associated and cell cycle-associated responses during regeneration

**Fig. 4:**
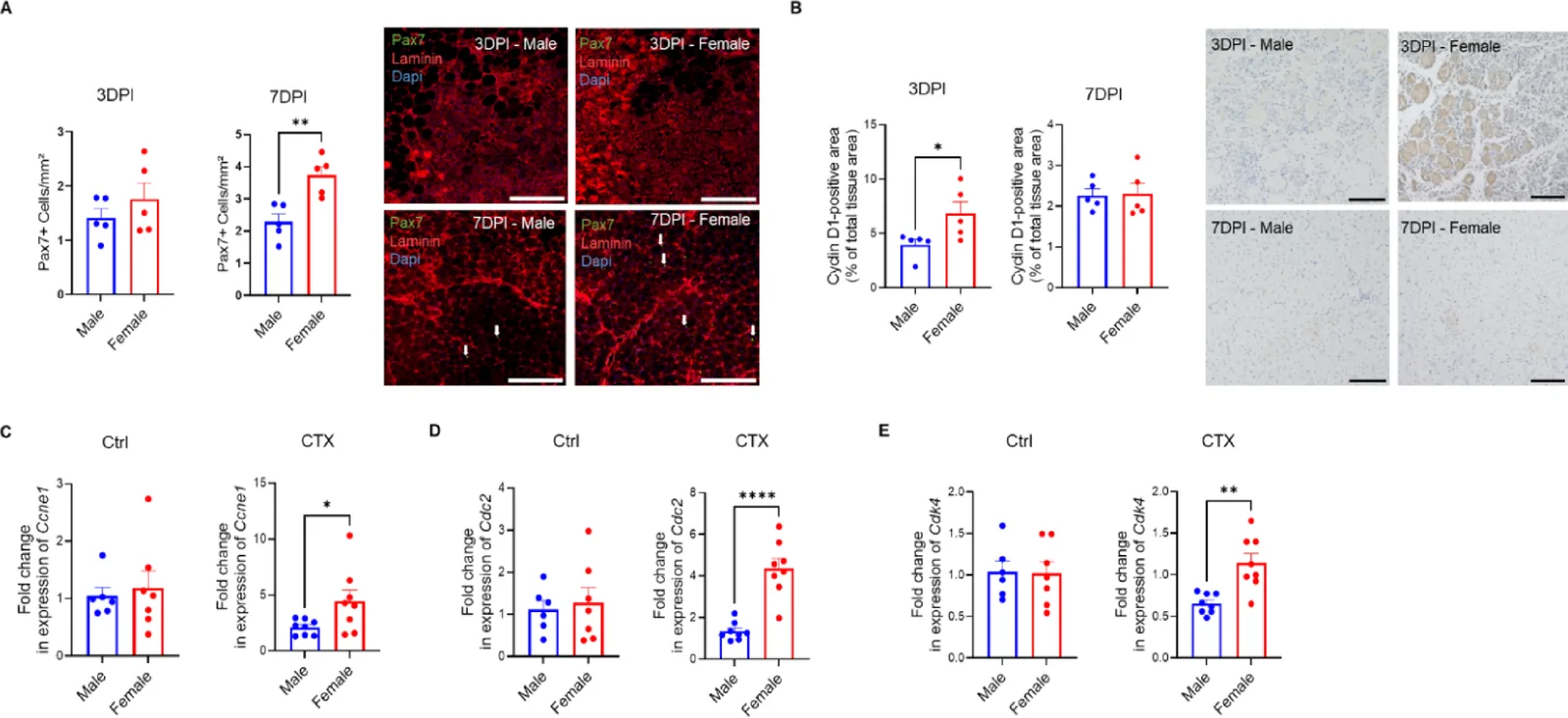
Sex-dependent satellite cell-associated and cell cycle-associated responses during regeneration. (**A**) Quantification of Pax7-positive cells and representative immunofluorescence images of Pax7 (green), laminin (red), and DAPI (blue) in regenerating tibialis anterior muscles at 3- and 7-days post-injury (DPI). White arrows indicate Pax7-positive satellite cells. (**B**) Quantification of Cyclin D1-positive area and representative immunohistochemical images of Cyclin D1 staining at 3 and 7 DPI. (**C**–**E**) Relative mRNA expression of *Ccne1* (**C**), *Cdc2* (**D**), and *Cdk4* (**E**) in male and female mice following cardiotoxin (CTX)-induced injury. Gene expression was normalized to *B2m* and expressed as fold change relative to the corresponding control group using the 2^ − ΔΔCt method. Data are presented as mean ± SEM. For immunohistochemical analyses (**A** and **B**), n = 5 per group. For qPCR analyses (**C**–**E**), n = 6–8 per group. Statistical significance was determined using an unpaired Student’s t-test. *P* < 0.05 was considered statistically significant. Scale bars = 100 μm.

To determine whether the observed transcriptional differences were accompanied by changes at the cellular level, Pax7 immunofluorescence and Cyclin D1 immunohistochemistry were performed. Pax7-positive cells were comparable between sexes at 3 DPI but were significantly more abundant in female mice at 7 DPI (Fig. 4A). Similarly, Cyclin D1-positive area was significantly greater in females at 3 DPI, whereas no significant sex difference was observed at 7 DPI (Fig. 4B). To further evaluate cell cycle-associated responses, expression of Ccne1, Cdc2, and Cdk4 was quantified. All three genes exhibited significantly greater expression in female mice following CTX injury compared with males (Fig. 4C–E). Together, these findings indicate that enhanced activation of the regenerative transcriptional program in female mice is accompanied by greater satellite cell-associated and cell cycle-associated responses during regeneration.

Because inflammatory signaling is closely linked to satellite cell function and tissue remodeling, we next characterized inflammatory responses following CTX-induced injury.

### Sex-dependent inflammatory responses during skeletal muscle regeneration

**Fig. 5:**
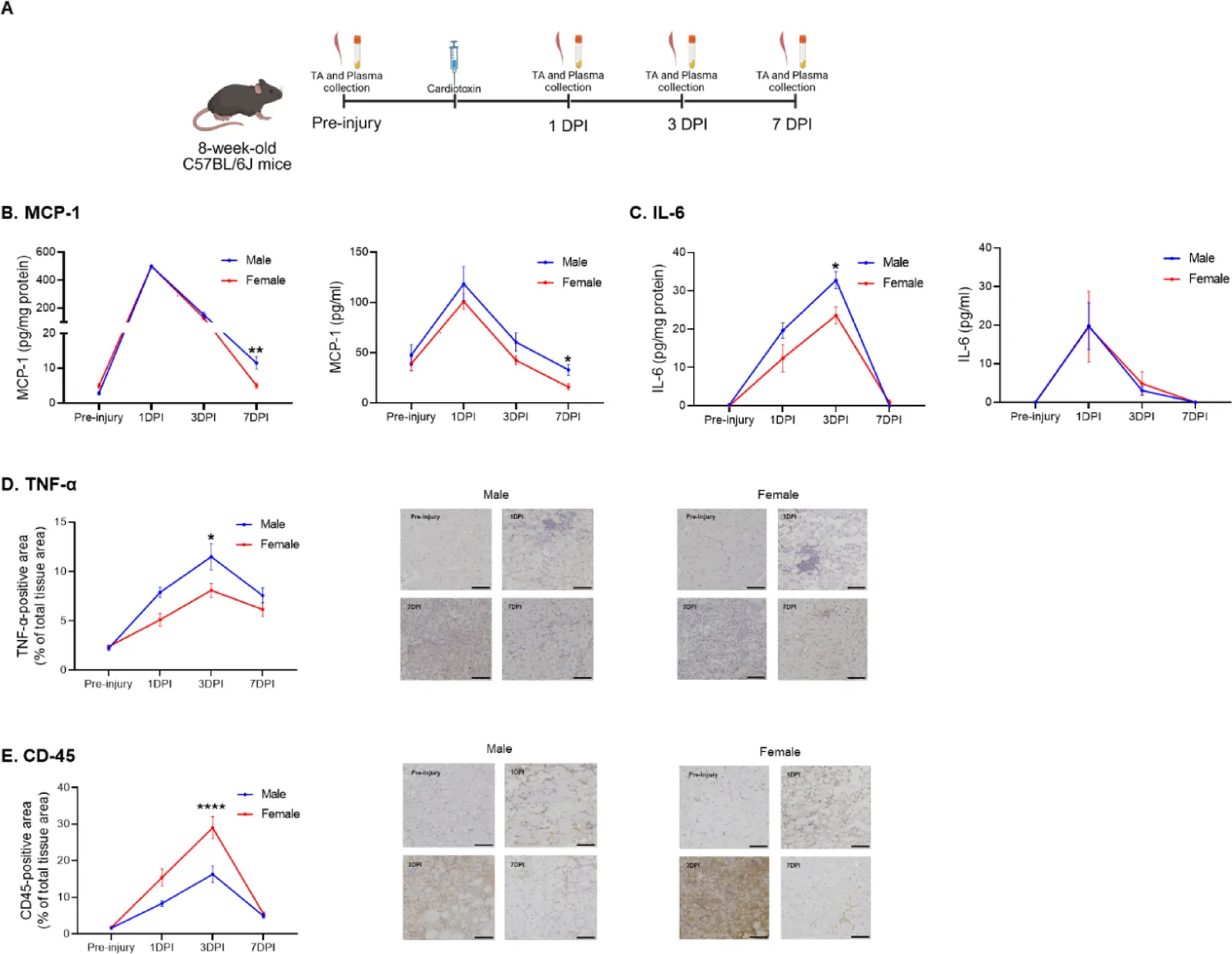
Sex-dependent inflammatory responses during skeletal muscle regeneration. (**A**) Experimental design showing plasma and tibialis anterior (TA) muscle collection at pre-injury and 1, 3, and 7 days post-injury (DPI) following cardiotoxin (CTX) injection. (**B**) TA muscle (left) and Plasma (right) MCP-1 levels in male and female mice across the indicated time points. Plasma MCP-1 concentrations are expressed as pg/mL, whereas muscle MCP-1 concentrations were normalized to total protein and expressed as pg/mg protein. (**C**) TA muscle (left) and Plasma (right) IL-6 levels in male and female mice across the indicated time points. Plasma IL-6 concentrations are expressed as pg/mL, whereas muscle IL-6 concentrations were normalized to total protein and expressed as pg/mg protein. Cytokine concentrations were measured using ELISA. (**D**) Quantification of TNF-α-positive area and representative immunohistochemical images of TNF-α staining in TA muscle collected at pre-injury and 1, 3, and 7 DPI. (**E**) Quantification of CD45-positive area and representative immunohistochemical images of CD45 staining in TA muscle collected at pre-injury and 1, 3, and 7 DPI. Positive staining is presented as the percentage of the total tissue area. Data are presented as mean ± SEM. For ELISA analyses (**B** and **C**), n = 7 mice per group. For immunohistochemical analyses (**D** and **E**), n = 5 mice per group. Statistical significance was determined using unpaired two-tailed Student’s t-test for comparisons between male and female mice at each time point. *p < 0.05, **p < 0.01, ***p < 0.001, ****p < 0.0001. Scale bars = 100 μm.

Inflammatory signaling plays an important role in skeletal muscle regeneration, thus, we next examined systemic and local inflammatory responses following CTX-induced injury. MCP-1 and IL-6 levels were measured in plasma and tibialis anterior (TA) muscle before injury and at 1, 3, and 7 days post-injury (DPI) (Fig. 5A). MCP-1 levels in TA muscle increased rapidly following CTX injury, peaked at 1 DPI, and gradually declined thereafter (Fig. 5B). Although MCP-1 levels were comparable between sexes at 1 and 3 DPI, male mice exhibited significantly higher MCP-1 levels than female mice at 7 DPI. Plasma MCP-1 showed a similar temporal pattern and remained significantly higher in males at 7 DPI. Muscle IL-6 levels reached their highest levels at 3 DPI and were significantly greater in male mice than in female mice (Fig. 5C). In contrast, plasma IL-6 peaked at 1 DPI and declined thereafter without significant differences between sexes.

To further evaluate local inflammatory responses, TNF-α and CD45 immunohistochemistry was performed on TA muscle sections collected before injury and at 1, 3, and 7 DPI. TNF-α-positive area increased following CTX injury in both sexes and peaked at 3 DPI, with significantly greater staining observed in male mice (Fig. 5D). In contrast, CD45-positive area also peaked at 3 DPI but was significantly greater in female mice (Fig. 5E).

Together, these findings indicate that inflammatory responses differ between males and females during skeletal muscle regeneration.

### Sex-dependent differences in tissue remodeling during skeletal muscle regeneration

**Fig. 6:**
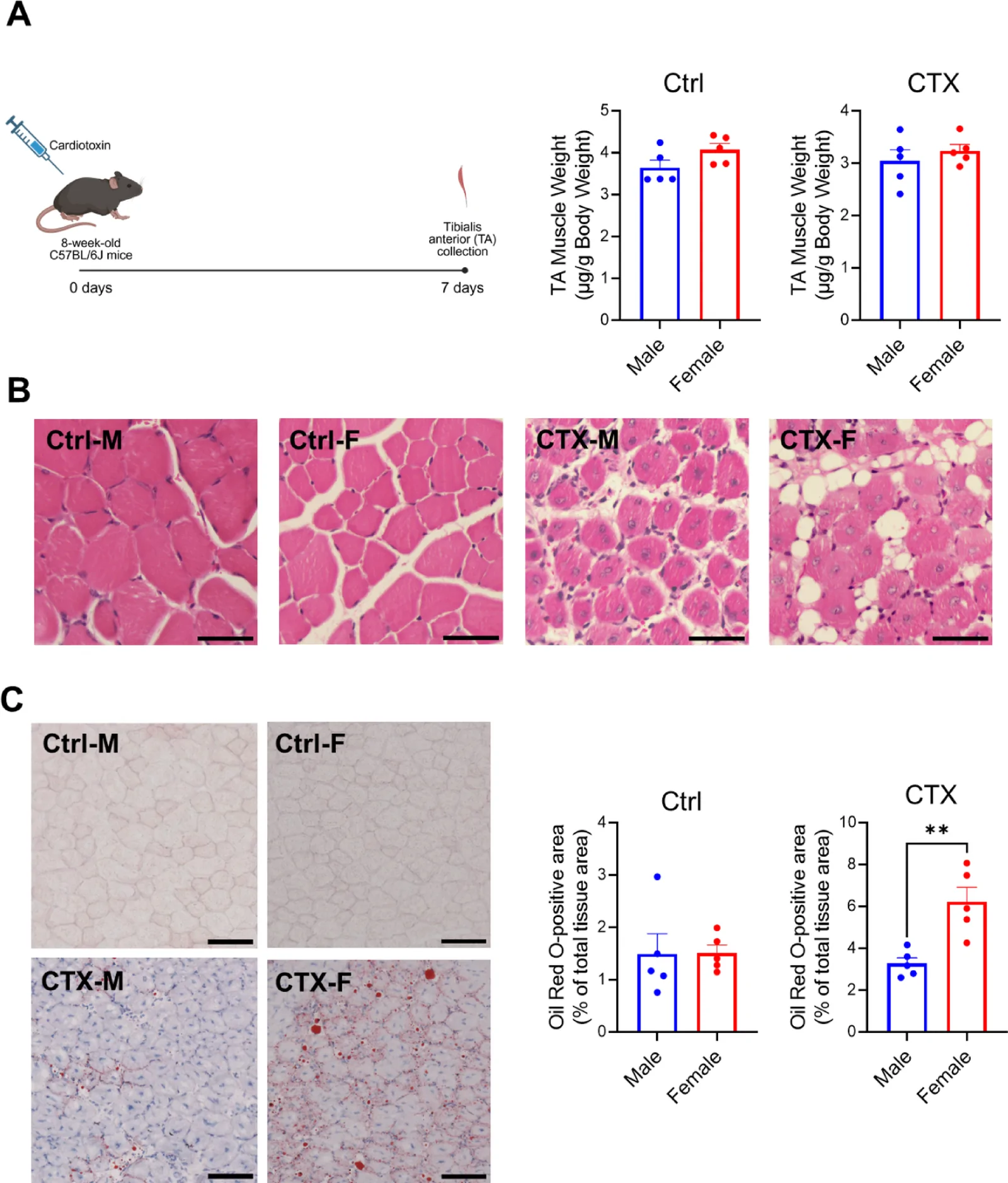
Sex-dependent differences in tissue remodeling during skeletal muscle regeneration. (**A**) Experimental design and quantification of tibialis anterior (TA) muscle weight normalized to body weight in control and CTX-injured male (M) and female (F) mice at 7 days post-injury (DPI). Data are presented as mean ± SEM. (**B**) Representative hematoxylin and eosin (H&E)-stained cross-sections of TA muscle from control and CTX-injured male and female mice at 7 DPI. Scale bar = 50 μm. (**C**) Representative Oil Red O-stained cross-sections and quantification of Oil Red O-positive area (% of total tissue area) in TA muscle from control and CTX-injured male and female mice at 7 DPI. Data are presented as mean ± SEM. Scale bar = 100 μm. Statistical significance was determined using an unpaired two-tailed Student’s t-test.

To determine whether the observed metabolic and molecular differences were reflected at the tissue level, muscle weight and histological analyses were performed at 7 DPI (Fig. 6). Normalized TA muscle weight did not differ significantly between males and females under either control or injured conditions (Fig. 6A). Hematoxylin and eosin staining demonstrated normal muscle morphology in control muscles from both sexes. Following CTX injury, both male and female muscles exhibited characteristic features of active regeneration, including centrally nucleated fibers and inflammatory cell infiltration (Fig. 6B). Female injured muscles also exhibited greater lipid accumulation than male muscles. To quantify this observation, Oil Red O staining was performed. Female mice showed significantly greater Oil Red O-positive area than male mice at 7 DPI, whereas no significant difference was observed under control conditions (Fig. 6C).

Because centrally nucleated fibers (CNFs) are a well-established marker of regenerating myofibers, CNF percentage was quantified as an additional indicator of regeneration. Although both sexes exhibited substantial numbers of regenerating fibers, no significant difference in CNF percentage was observed between males and females at 7 DPI (Supplementary Fig. S2). Together, these findings indicate that early tissue remodeling differs between males and females despite similar progression of myofiber regeneration. We next determined whether these early differences influenced the overall regenerative outcome.

### Comparable structural recovery despite distinct regenerative responses

**Fig. 7:**
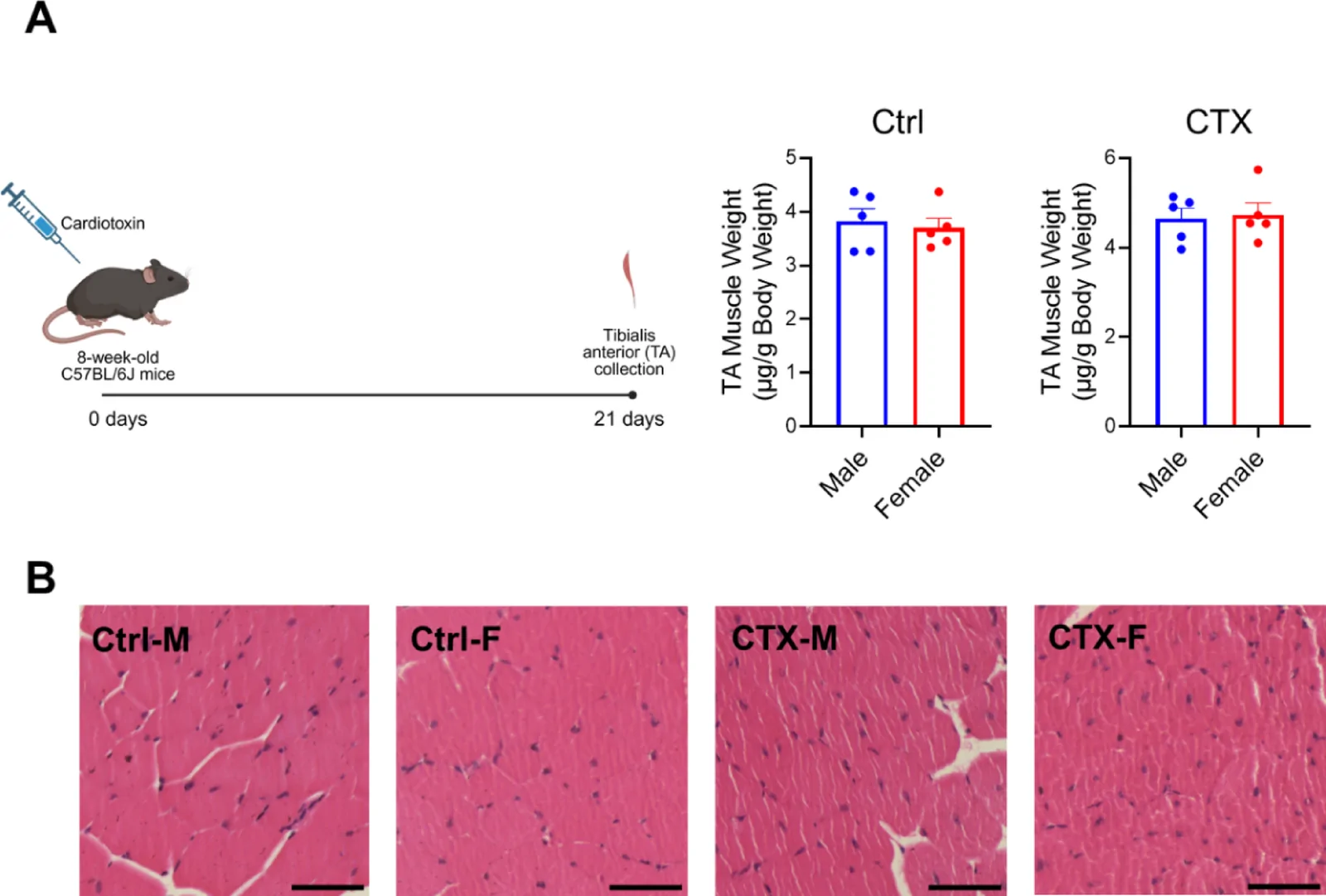
Comparable structural recovery despite distinct regenerative responses. (**A**) Experimental design and quantification of tibialis anterior (TA) muscle weight normalized to body weight in control and CTX injured male (M) and female (F) mice at 21 days post injury (DPI). Data are presented as mean ± SEM. (**B**) Representative hematoxylin and eosin (H&E)-stained cross-sections of TA muscle from control and CTX-injured male and female mice at 21 DPI. Scale bar, 50 µm. Statistical significance was determined by unpaired two-tailed Student’s t-test.

To determine whether the early sex-dependent differences observed during regeneration affected the final regenerative outcome, TA muscles were evaluated at 21 DPI. Normalized TA muscle weight did not differ significantly between males and females following CTX injury (Fig. 7A). Histological analysis further demonstrated restoration of muscle architecture in both sexes, with well-organized myofibers and minimal residual pathological changes (Fig. 7B). These findings indicate that although males and females exhibit distinct metabolic, molecular, inflammatory, and tissue remodeling responses during the early phase of regeneration, both sexes ultimately achieve comparable structural recovery following CTX-induced injury.

## Discussion

In the present study, we found that biological sex was associated with differences in metabolic remodeling, regenerative gene expression, satellite cell-associated responses, inflammatory responses, and tissue remodeling during skeletal muscle regeneration. Although males and females exhibited distinct biological responses during the early phase of regeneration, both sexes ultimately achieved comparable structural recovery following CTX-induced injury. Overall, our findings suggest that males and females undergo skeletal muscle regeneration through distinct biological responses during the early phase of regeneration while achieving a similar structural outcome.

Metabolic remodeling is an important component of skeletal muscle regeneration because metabolic pathways provide both energy and biosynthetic intermediates required for tissue regeneration^12^. In the present study, PCA and pathway enrichment analyses showed that metabolic profiles became more distinct between males and females following CTX-induced injury, with glycolysis/gluconeogenesis identified as the most significantly altered pathway. Consistent with these findings, several glycolytic and TCA cycle metabolites, including lactate, fructose-1,6-bisphosphate, 3-phosphoglycerate, succinate, and 2-hydroxyglutarate^13–19^, were present at higher levels in male mice than female mice at 7 DPI. In addition, methionine, proline, and asparagine also differed between sexes, indicating that the observed metabolic differences extended beyond central carbon metabolism^20–23^. Despite these metabolic differences, female mice exhibited greater expression of regenerative genes and satellite cell-associated markers. These findings suggest that sex-dependent metabolic remodeling is associated with, but does not necessarily predict, regenerative activity.

Regeneration of skeletal muscle depends on activation of myogenic regulatory factors that control satellite cell activation and myogenic progression^24–27^. Female mice exhibited significantly greater expression of Pax7, Myf5, and Myod1 than male mice at 7 DPI, indicating a stronger regenerative transcriptional response during the intermediate stage of regeneration. These transcriptional changes were accompanied by increased numbers of Pax7-positive cells and greater expression of Cyclin D1, Ccne1, Cdc2, and Cdk4, suggesting enhanced satellite cell-associated and cell cycle-associated responses in female mice. Previous studies have shown that metabolic regulation influences satellite cell function during regeneration^28–32^. Our findings are consistent with these observations and further suggest that sex-dependent metabolic differences are associated with differences in regenerative responses following skeletal muscle injury.

Inflammatory responses play an essential role in skeletal muscle regeneration by regulating immune cell recruitment, satellite cell activity, and tissue remodeling^33,34^. In the present study, local inflammatory responses differed between males and females throughout regeneration. Male mice exhibited greater MCP-1, IL-6, and TNF-α responses, whereas female mice showed greater CD45-positive cell infiltration. Although the functional significance of these differences remains to be determined, the findings suggest that males and females establish different inflammatory environments during regeneration. Similar sex-dependent inflammatory responses have been reported in both skeletal muscle and other tissues^35,36^.

Histological findings were consistent with the molecular analyses. Female mice exhibited greater lipid accumulation at 7 DPI, as demonstrated by both H&E morphology and quantitative Oil Red O staining. Transient lipid accumulation has been reported during skeletal muscle regeneration and may reflect temporary changes in lipid metabolism rather than persistent pathological remodeling^37,38^. In contrast, the percentage of centrally nucleated fibers did not differ significantly between sexes, indicating comparable progression of myofiber regeneration. Furthermore, both sexes exhibited similar restoration of muscle architecture by 21 DPI. These findings suggest that early differences in tissue remodeling do not necessarily alter the overall regenerative outcome.

Overall, the present findings suggest that metabolic regulation is associated with multiple biological processes during skeletal muscle regeneration, including regenerative gene expression, satellite cell-associated responses, inflammatory responses, and tissue remodeling. Although the present study does not establish causal relationships among these processes, the data provide a comprehensive characterization of sex-dependent regenerative responses following CTX-induced muscle injury.

Several limitations should be considered. First, this study examined only young adult C57BL/6 J mice, and therefore the findings may not be directly applicable to aging or disease-associated muscle regeneration. Second, circulating sex hormone levels were not measured, preventing direct evaluation of hormone-dependent effects^39–41^. Finally, the present study was designed to characterize sex-dependent regenerative responses rather than establish causal mechanisms. Future studies using single-cell transcriptomics, spatial metabolomics, and functional approaches will be important for determining how metabolism contributes to skeletal muscle regeneration.

## Conclusion

In conclusion, biological sex was associated with differences in metabolic remodeling, regenerative gene expression, satellite cell-associated responses, inflammatory responses, and tissue remodeling during skeletal muscle regeneration. Although males and females ultimately exhibited comparable structural recovery following CTX-induced injury, the regenerative process differed substantially during the early phase after injury. These findings provide additional insight into sex-dependent skeletal muscle regeneration and serve as a foundation for future studies investigating how metabolism contributes to regenerative responses.

## Supplementary Information

Below is the link to the electronic supplementary material.

Supplementary Material 1.

Supplementary Material 2.

## Data Availability

All metabolomics data supporting the findings of this study are provided in the paper and its Supplementary Information. Additional data supporting the findings of this study are available from the corresponding author upon reasonable request.

## Abbreviations

2-HG: 2-Hydroxyglutarate
3-PG: 3-Phosphoglycerate
Ccne1: Cyclin E1
Cdk4: Cyclin-dependent kinase 4
Cdc2: Cell division cycle 2 (CDK1)
CNF: Centrally nucleated fiber
CTX: Cardiotoxin
DPI: Days post injury
F-1,6-BP: Fructose-1,6-bisphosphate
H&E: Hematoxylin and eosin
IF: Immunofluorescence
IHC: Immunohistochemistry
IL-6: Interleukin-6
LC–MS: Liquid chromatography–mass spectrometry
MCP-1: Monocyte chemoattractant protein-1
PCA: Principal component analysis
TA: Tibialis anterior
