# Supplementary material for: Sex differences in metabolic remodeling and skeletal muscle regeneration following cardiotoxin-induced injury
Source: Sci Rep. 2026 Jul 13;16:32162. doi: 10.1038/s41598-026-62000-9 (PMC13661242; doi:10.1038/s41598-026-62000-9)
Supplement: Supplementary file 1 [file 41598_2026_62000_MOESM2_ESM.docx]

**Supplementary Figure S1. Pathway enrichment analysis of differentially abundant metabolites following CTX-induced skeletal muscle injury.**

Bubble plot showing the top enriched metabolic pathways identified by pathway enrichment analysis of significantly altered metabolites following cardiotoxin (CTX)-induced skeletal muscle injury. The x-axis represents the −log10(P-value), bubble size indicates the enrichment ratio, and bubble color corresponds to the pathway P-value. Glycolysis/gluconeogenesis was identified as the most significantly enriched metabolic pathway.

**Supplementary Figure S2. Whole transverse muscle sections and quantification of centrally nucleated fibers following CTX-induced injury.**

(A) Experimental design and quantification of centrally nucleated fibers (CNFs) in tibialis anterior (TA) muscles collected at 7 days post-injury (DPI). CNFs were manually quantified and expressed as a percentage of total myofibers. Data are presented as mean ± SEM (n = 4-5 per group).

(B) Representative whole transverse H&E-stained TA muscle sections from male and female control and CTX-injured mice. Scale bar = 1 mm.

| **Gene** | **Forward Primer (5'→3')** | **Reverse Primer (5'→3')** |
| --- | --- | --- |
| Myog | GAGACATCCCCCTATTTCTACCA | GCTCAGTCCGCTCATAGCC |
| Myf5 | AAGGCTCCTGTATCCCCTCAC | TGACCTTCTTCAGGCGTCTAC |
| Myod1 | AAAAGCGCAGGTCTGGTGAG | CCACTCCGGGACATAGACTTG |
| Pax7 | TCTCCAAGATTCTGTGCCGAT | CGGGGTTCTCTCTCTTATACTCC |
| Ccne1 | GCGAGGATGAGAGCAGTTC | AAGTCGTGTGCCAAGTAGAAC |
| Cdk4 | GTGCCAGAGATGGAGGAGTC | TTGTGCAGGTAGGAGTGCTG |
| Cdc2 | AGCTCTGGGGACTCCTAACA | TCCATCCAGAGGGCTACATC |
| B2m | TTCTGGTGCTTGTCTCACTGA | CAGTATGTTCGGCTTCCCATTC |

**Supplementary Table S1. Primer sequences used for quantitative real-time PCR (qRT-PCR) analysis.**

Forward and reverse primer sequences (5′–3′) used for the amplification of Myog, Myf5, Myod1, Pax7, Ccne1, Cdk4, Cdc2 and B2m transcripts in mouse skeletal muscle samples.
